# Do All Fractures in the Ankylotic Spine Really Require Surgical Intervention?

**DOI:** 10.3390/jcm14155599

**Published:** 2025-08-07

**Authors:** Moshe Stavsky, Elad Harats, Ahmad Sharabati, Amjad Hamad, Harel Arzi, Bilal Qutteineh, Yair Barzilay

**Affiliations:** Spine Unit, Department of Orthopedic Surgery, Shaare Zedek Medical Center, Faculty of Medicine, Hebrew University, Jerusalem 91120, Israel; moyshi@gmail.com (M.S.); haratselad@gmail.com (E.H.); ahmad.bi.sharabati@gmail.com (A.S.); dramjadha@gmail.com (A.H.); dr.harelarzi@gmail.com (H.A.); drbilal87@gmail.com (B.Q.)

**Keywords:** ankylotic spine, spine fracture, extension-type fracture, DISH (Diffuse Idiopathic Skeletal Hyperostosis) conservative treatment

## Abstract

**Background**: Patients with ankylotic spines suffering from vertebral column fractures are frequently operated on to maintain spinal stability and prevent secondary displacement and nerve damage. The aim of this study was to identify a subset of patients that may be treated non-operatively, thus avoiding operative complications in this group of patients. **Methods**: Extension-type injuries in patients with DISH (diffuse idiopathic skeletal hyperostosis) not involving the posterior elements of the spine comprised the study group. **Results**: Twenty two extension fractures occurred in 21 patients with DISH in SZMC (Shaare Zedek Medical Cente) between 2014–2025. All patients were treated non-operatively. Patients were allowed free mobilization, and no orthosis was used. The only limitation was keeping the bed inclined to 20–30 degrees to prevent extension at the fracture site. All fractures healed uneventfully, and no patient required late surgical intervention, and no neurological complications were noted. **Conclusions**: Patients with DISH who sustain extension-type injuries of the thoracolumbar spine, with no involvement of the posterior elements, may be treated non-operatively, with good results.

## 1. Introduction

Ankylosing spinal disorders (ASDs), primarily diffuse idiopathic skeletal hyperostosis (DISH) and ankylosing spondylitis (AS), are characterized by pathological ossification of spinal ligaments and entheses, leading to a rigid and brittle vertebral column. This rigidity renders the spine highly susceptible to fractures, even from minor trauma [1].

Diffuse idiopathic skeletal hyperostosis (DISH) is a systemic musculoskeletal disorder characterized by non-inflammatory ossification of ligaments and tendons, predominantly affecting the anterior and lateral aspects of the vertebral column. This process often results in continuous, bridge-like bony growths across several adjacent spinal segments [2]. While primarily affecting the spine, ossification can also occur at peripheral entheses, such as in the shoulders, elbows, knees, and calcanei [3].

The extensive ossification in DISH creates long, rigid segments in the spine, acting as a long lever arm that amplifies forces during trauma. This, coupled with reduced spinal flexibility [4], concentrates stress at fracture sites, leading to a higher risk of unstable fractures with potential neurological compromise or deformity. The significant concern for secondary displacement, catastrophic neurological complications, and compressive epidural hematoma [5] typically drives an aggressive management approach, often involving strict bed rest and early surgical fixation.

Guidelines published by the German Spine Society [6] recommend surgical treatment for every patient with a fracture involving the ankylotic spine. Similarly, the AO Spine Classification System designates extension-type injuries (specifically B3 fractures) as requiring surgical intervention [7,8]. However, surgical intervention in this patient population is often challenging due to advanced age, numerous comorbidities, and inherent frailty, leading to high rates of complications and mortality [9,10].

An extension-type spinal fracture is a common injury pattern in ankylosed spines, characterized by a hyperextension mechanism involving distractive forces predominantly on the anterior column of the spine. These injuries often result from a combination of hyperextension and axial loading, leading to fracture patterns that are exacerbated by further extension but reduced by flexion.

In the thoracolumbar region, extension injuries typically manifest as oblique fracture lines traversing the vertebral body, often accompanied by the widening of the facet joints [11]. While historically classified as unstable and managed surgically, the recent literature suggests that certain subsets of these fractures, particularly those without posterior element involvement, might be amenable to non-operative treatment.

The aim of this study was to identify a subset of patients with fractures of the ankylotic spine who do not require surgical intervention and to offer a treatment protocol—thus avoiding the high operative risk. This group is the patients with DISH, without ankylosing of the posterior elements, who sustain extension-type injuries of the anterior elements of the thoracolumbar spine, with no involvement of the posterior elements.

## 2. Methods

This retrospective cohort study was conducted at Shaare Zedek Medical Center (SZMC) and included patients treated between January 2014 and March 2025. This study received approval from the Institutional Review Board Committee of SZMC (approval number 0239-23).

### 2.1. Patient Identification and Inclusion Criteria

Patient medical records were systematically screened for patients hospitalized with spinal fractures using relevant International Classification of Diseases (ICD) codes. The primary inclusion criteria were patients diagnosed with diffuse idiopathic skeletal hyperostosis (DISH) who presented with extension-type fractures of the thoracolumbar spine. Key exclusion criteria included fractures involving the cervical spine; fractures requiring immediate surgical intervention due to instability or neurological deficit; extension-type fractures with clear evidence of posterior element involvement (e.g., disruption of the posterior ligamentous complex and facet joint fracture/subluxation); and patients with ankylosing spondylitis (AS) or other inflammatory spondyloarthropathies to ensure a homogenous study population focused on DISH. A total of 21 patients meeting these criteria with 22 thoracolumbar extension-type fractures were identified and comprised the study group.

### 2.2. Treatment Protocol

An internal, non-operative treatment protocol was consistently applied to all included patients. Upon admission, comprehensive clinical and neurological examinations were performed. All patients underwent computed tomography (CT) scans of the affected spinal region. These scans were meticulously reviewed by experienced spine surgeons to rule out involvement of the posterior elements of the spine. In cases where physical examination revealed tenderness over the posterior elements, magnetic resonance imaging (MRI) scans were performed to definitively assess for injury to the posterior ligamentous complex (PLC). Patients were initially placed on bed rest with the bed inclined to 20–30 degrees. This specific inclination was maintained to promote gravity-assisted reduction and prevent hyperextension at the fracture site, thereby ensuring constant contact between fracture surfaces. Under close supervision of the attending spine surgeon, patients were gradually allowed to sit up, and then ambulate, provided no significant pain or signs of instability were observed. No external bracing or orthosis was utilized at any point during the treatment protocol. Patients were mobilized freely, typically with the aid of walkers or other assistive devices as needed. Standing radiographs (X-rays) of the spine were performed immediately upon ambulation to confirm the absence of fracture displacement. Subsequent standing X-rays were conducted at 1, 3, and 6 weeks post-injury in the outpatient spine clinic to monitor the fracture healing progress. Follow-up continued until definitive radiographic evidence of fracture healing was observed.

### 2.3. Data Collection and Outcome Measures

Demographic data collected included age, sex, and the mechanism of injury. Clinical data included the level of injury and the initial neurological status. Primary outcome measures included radiographic evidence of fracture healing, the need for late surgical intervention, and the occurrence of neurological complications. Secondary outcomes included any other reported complications (e.g., pneumonia). In cases of missing or incomplete data, telephone interviews with patients or their families were conducted to obtain the necessary information.

## 3. Results

This study included 21 patients with a total of 22 thoracolumbar extension-type spinal fractures, as one patient suffered from two distinct fractures at different times. All patients had a confirmed diagnosis of diffuse idiopathic skeletal hyperostosis (DISH). The mean follow-up period for the cohort was 11 months, ranging from 1.5 to 72 months, with follow-up extending until radiographic evidence of fracture healing was confirmed.

The demographic and injury characteristics of the study population are summarized in Table 1. The average age was 80.4 ± 8.9 years (the mean ± the standard deviation). The cohort predominantly consisted of females, with 14 females and nine males. The mechanisms of injury were falling from standing height in 16 patients (76%), MVA in 3 patients (14%), and other mechanisms in 2 patients (10%). The mean follow-up was 11 months (range 1.5–72).

A key finding of this study was that all 22 fractures successfully healed uneventfully under the described non-operative protocol. An elderly and frail patient who sustaining extension type injury in DISH spine twice is shown in Appendix A. No neurological deterioration was observed in any patient throughout their treatment and follow-up period. Furthermore, no patient required late surgical intervention for fracture instability or non-union. Two patients, however, experienced unrelated deaths due to pneumonia at 3 and 4 months following their respective injuries. It is important to note that both of these patients’ fractures had healed uneventfully prior to their demise.

## 4. Discussion

Management of spinal fractures in patients with ankylosing spinal disorders (ASDs), particularly diffuse idiopathic skeletal hyperostosis (DISH), presents a complex and frequently debated clinical scenario. The inherent rigidity and often concomitant osteoporosis render these spines highly susceptible to fractures even from low-energy trauma [6]. While current guidelines predominantly advocate for surgical intervention, emerging evidence, supported by our findings, suggests that specific subsets of these injuries may be safely and effectively managed non-operatively.

### 4.1. Current Guidelines and Surgical Outcomes

Current consensus guidelines from the German Spine Society [6] classify fractures involving ankylotic spines as inherently unstable, generally recommending surgical fixation to prevent secondary displacement and neurological deterioration. Similarly, the widely utilized AO Spine Classification System [7] designates extension-type injuries (B3 fractures) as typically unstable, for which surgery is advised [8]. The TLICS system [12] also considers distraction-type injuries as potentially unstable; however, when the posterior ligamentous complex remains intact and no neurological deficit exists, the decision for management may be left to the treating surgeon’s discretion. Despite these recommendations, reports on surgical treatment frequently highlight substantial and concerning complication rates in this frail and elderly patient population. Cirillo et al. (2022) [13] described a cohort of 13 patients with DISH or AS undergoing surgery, noting a high complication rate of 63%, which included surgical site infections and mortality. Similarly, Balling and Weckbach (2015) [14] observed alarming rates of medical complications (65%) and mortality (17.4%) among surgically treated patients. These high complication and mortality rates in surgically managed cohorts underscore the significant benefits of identifying patients suitable for non-operative treatment, thereby avoiding the inherent risks associated with surgical intervention in this vulnerable population.

### 4.2. Evidence for Non-Operative Management in the Literature

In contrast to the high surgical risks, several studies, including our own, have reported successful conservative management of fractures in DISH patients. Poul et al. (2022) [15] conducted one of the largest series to date, managing 21 patients with DISH and extension-type fractures non-operatively using a thoracolumbosacral orthosis (TLSO). Critically, all their patients completed treatment without neurological deterioration, fracture displacement, or the need for delayed surgery, demonstrating the feasibility of conservative treatment. Further support for this approach comes from case reports by Kuroki et al. (2021) [16] and Saita et al. (2019) [17], who similarly documented uneventful healing under bracing in smaller series.

### 4.3. Our Unique Non-Operative Protocol and Its Advantages

The unique and central aspect of our study lies in the successful non-operative management of anterior element extension-type fractures in DISH patients without any form of external bracing. Our specific treatment protocol required patients to be positioned with their beds elevated at 20–30 degrees to maintain constant contact between fracture surfaces. This gravity-assisted reduction, combined with the inherently large fracture surfaces and the intact posterior tension band, allowed for effective fracture reduction and maintained stability throughout the healing process. As a result, all 22 fractures in our series of 21 patients achieved complete healing without neurological deficits or secondary displacement. This approach significantly reduces the patient burden associated with orthoses while still mitigating the high operative risks. Our study demonstrates that this protocol is feasible, and complications directly from the non-operative treatment itself are rare. Moreover, the absence of secondary displacement or neurological deterioration further validates its safety and efficacy.

### 4.4. Implications and Patient Selection

**Figure 1:**
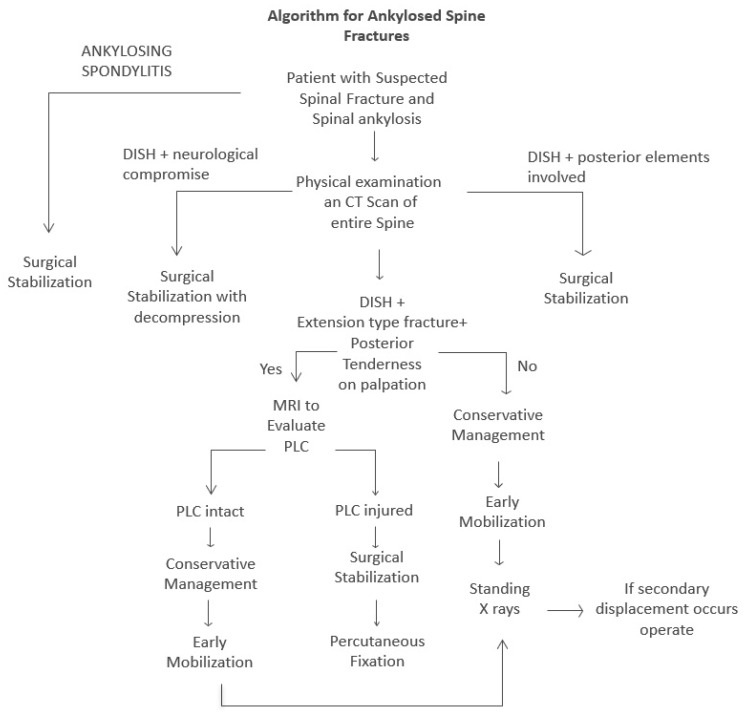
Proposed management algorithm for spinal fractures in patients with ankylotic spines.

It is critical to differentiate these isolated anterior element extension-type fractures, which were the focus of our study, from the more unstable three-column fractures or those with posterior element involvement. These more complex injuries are typical in patients with ankylosing spondylitis or more severe DISH, and for such highly unstable fractures, surgical stabilization remains the standard of care. Our findings challenge the broad generalization that all extension-type fractures in ankylosed spines require surgery, highlighting the importance of nuanced patient selection and detailed radiological assessments, particularly for posterior ligamentous complex integrity. This study provides compelling evidence that a re-evaluation of current treatment algorithms for this specific fracture pattern in DISH patients may be warranted, potentially encouraging the integration of conservative treatment into existing management strategies. Our proposed management algorithm for patients with ankylotic spines presenting with spinal fractures is presented in Figure 1.

### 4.5. Limitations

While no neurological complications were observed in our cohort, two patients died in the months following the fracture from pneumonia unrelated to the trauma. These complications were not directly related to fracture stability or failure of conservative management but rather reflect the underlying comorbidities prevalent in this patient population. Our study is not without limitations. Its retrospective design inherently carries risks of selection bias and incomplete data collection. The relatively small sample size of 21 patients, though notable for this specific and challenging fracture pattern, limits the generalizability of our findings. Furthermore, the absence of a direct comparison group (e.g., surgically treated patients within the same institution) precludes a definitive statement on superiority, although historical data from surgical cohorts strongly suggest the advantages of avoiding surgery. Future larger, prospective, multi-center studies with standardized protocols are needed to validate our findings and provide higher levels of evidence.

## 5. Conclusions

Our study demonstrates that isolated anterior element extension-type fractures in DISH patients, where the posterior elements remain intact, can be safely and successfully managed non-operatively, eliminating the need for external orthoses. The implementation of a strict bed-elevation protocol (20–30 degrees) proved effective in achieving fracture healing and preventing secondary complications often associated with surgical interventions in this frail population. While acknowledging the retrospective nature and limited sample size of this study, our findings suggest a re-evaluation of current guidelines for this specific fracture pattern. This highlights the potential for a non-operative approach to reduce morbidity in carefully selected patients. Future prospective, multi-center research is warranted to validate these findings and to inform the development of a more nuanced classification system that better guides treatment decisions for ankylotic spine fractures.

## Figures and Tables

**Table 1:** Demographics and location of extension-type fractures in DISH patients.

Age	Gender	Fracture Site	Complications
69	F	L1	No
71	F	T11	No
81	M	T6–T7	No
81	M	T10–T11	No
89	M	T12	No
89	F	L1	No
88	F	T10	No
67	F	T12	No
79	F	T7	No
85	F	T10 + T11	No
91	F	T8	No
88	M	T5	No
87	F	T7	No
82	M	L1	No
76	M	T8	No
55	F	T10	No
67	M	T10–T11	No
86	F	T11	No
85	F	T11	No
84	F	L1	No
83	M	T7	No
86	F	L1	No

**Figure A1:**
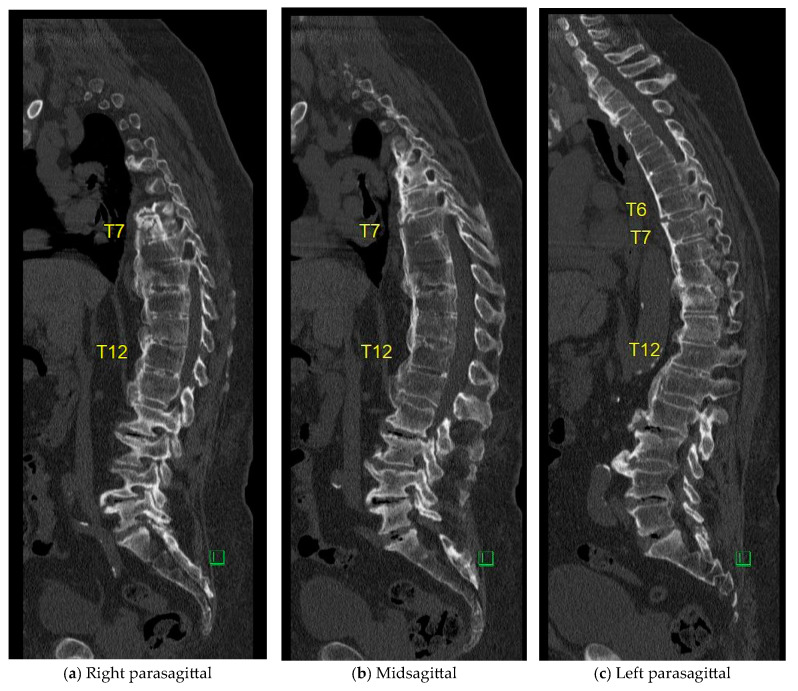
Parasagittal (**a**,**c**) and Mid-sagittal (**b**) CT reconstructions obtained one year following a T9–T10 extension-type injury and five days after a subsequent backward fall. A new extension-type injury at the T6–T7 level is identified. No involvement of the posterior elements is observed.

**Figure A2:**
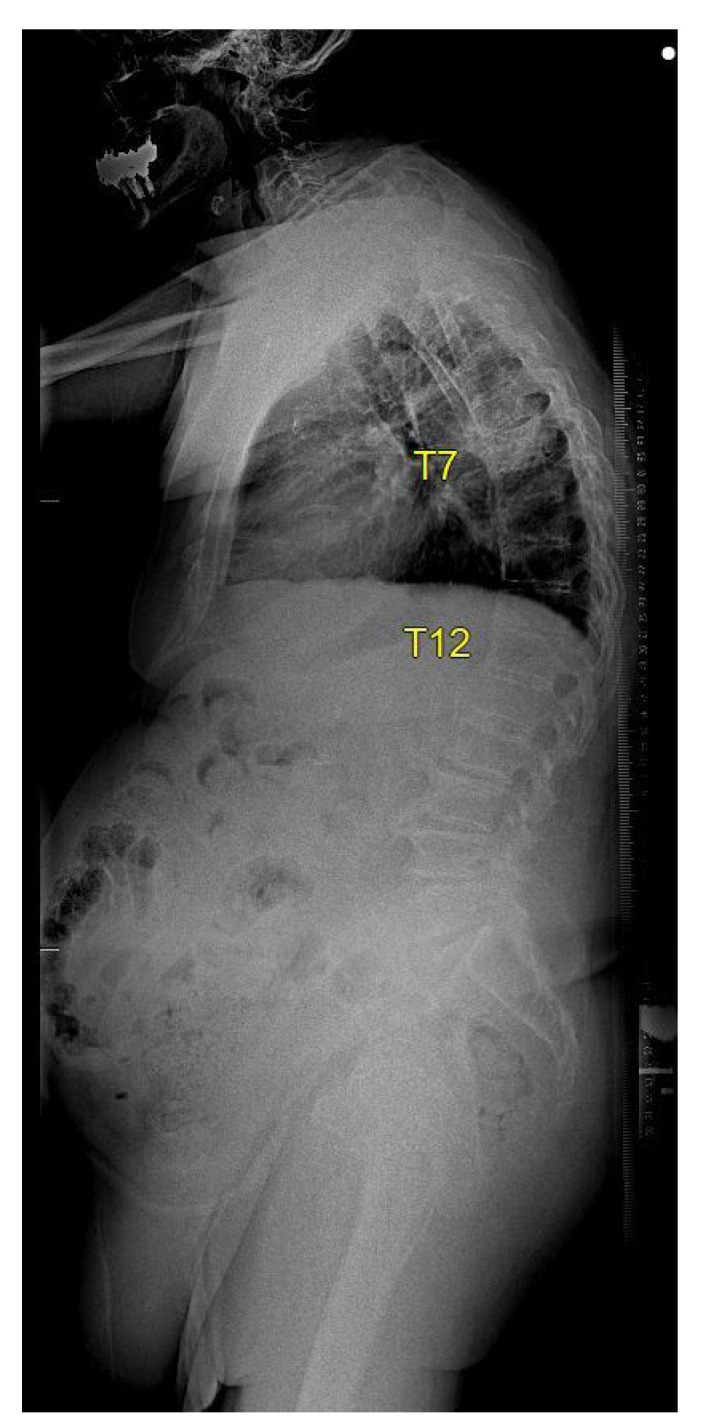
Standing X-ray performed 4 months post-fracture, demonstrating solid fracture healing at T6–T7.

## Data Availability

The datasets generated and analyzed during the current study are not publicly available due to them containing sensitive and private patient information that was collected retrospectively for research purposes, thereby precluding anonymization to a degree that would allow for open sharing while maintaining patient confidentiality. However, the data are available from the corresponding author upon reasonable request and with appropriate institutional ethics board approval. Requests for data should be directed to Yair Barzilay (barzilay@szmc.org.il).

## Appendix A

The case of an 81-year-old male, suffering from ischemic heart disease, atrial fibrillation, hypertension, morbid obesity, and gout, illustrates a unique treatment approach. He presented to the E.R. three days after a backward fall, having sustained an extension-type injury to T10–T11 a year and a half prior, which was treated non-operatively due to a very high operative risk. In the current fall, a new extension-type injury was identified at the T6–T7 level. He experienced back pain without radiating symptoms and had a long-standing foot drop but no new nerve deficit. His CT scans are shown in Figure A1. At a 4-month follow-up visit to the outpatient clinic, standing X-rays were performed, further confirming the stable healing of the fracture as shown in Figure A2.

The successful outcome in this specific case, particularly the complete healing of all three fractures (including prior conservatively managed fractures) with no sequelae, supports considering a non-operative approach in this unique group of patients. This result highlights the potential for successful conservative management even in complex cases.
